# Editorial: Advances and challenges in untargeted metabolomics

**DOI:** 10.3389/fmolb.2023.1097443

**Published:** 2023-02-10

**Authors:** Renata Wawrzyniak, Franciso Javier Ruperez, Joanna Barbara Godzień

**Affiliations:** ^1^ Department of Biopharmaceutics and Pharmacodynamics, Medical University of Gdansk, Gdansk, Poland; ^2^ Centre for Metabolomics and Bioanalysis (CEMBIO), Facultad de Farmacia, Universidad CEU San Pablo, CEU Universities, Madrid, Spain; ^3^ Metabolomics Laboratory, Clinical Research Centre, Medical University of Bialystok, Bialystok, Poland

**Keywords:** untargeted (global) metabolomics, sample preparation, analytical methods, data processing and analysis, metabolite identification, applications

Metabolomics is an emerging approach in the systems biology field. An untargeted strategy, which is not driven by any preliminary assumption, aims to define changes in the whole metabolome, which occur at the specific state in cells, tissues, or whole organism (Moco and Buescher, 2023). This allows to state new hypotheses and may direct the research into a new and often unexpected course. Despite the excellent advances that were made in the last decade, some improvements are still required at each stage of the untargeted metabolomics workflow.

The main aim of this Research Topic is to unveil novel techniques and tools in the field of untargeted metabolomics, focusing on their applications. The aspects covered by this papers Research Topic include the use of unconventional sample for untargeted metabolomics (Buszewska et al.), interesting analytical approaches for the sample measurement (Filipiak et al., Mojsak et al., Jensen-Kroll et al.), innovative data analyses (Shaver et al.), novel approaches increasing confidence in metabolite annotation (Barrero-Rodríguez et al., Traquete et al.) and applications (Małachowska et al., Lackner et al.).

Buszewska et al. have reported the results of study aimed to develop and optimize the sample preparation procedure for ejaculate samples. The optimized method was applied for the untargeted metabolomics of seminal fluid samples derived from prostate cancer patients. Finally, the metabolic signatures of seminal fluid, urine and plasma samples were determined with the use of two complementary analytical techniques: GC-EI-QqQ/MS and LC-ESI-TOF/MS and subsequently compared.

Filipiak et al. have investigated the clinical strains of *K. pneumonia* (KPN) isolated from bronchoalveolar lavage specimens collected from mechanically ventilated patients to reveal, whether bacterial volatile organic compounds (VOCs) observed in model experiments with reference strains could be relevant for wild pathogens as well. For analytical measurements, the headspace samples from bacteria cultures were collected at seven time points on multibed sorption tubes and analyzed by GC-MS to follow the dynamic changes in VOC concentrations. Altogether 32 VOCs were released by susceptible and 25 VOCs by resistant strain, amongst which 2-pentanone, 2-heptanone, and 2-nonanone were significantly higher for carbapenem-resistant KP.

Mojsak et al. have demonstrated an optimisation of plasma and serum preparation for the measurement of 75 microbiota-dependent metabolites (MDMs). Different solvents or solvent mixtures for MDMs extraction, various concentrations and volumes of derivatizing reagents as well as temperature programs for methoxymation and silylation, have been tested. Finally, the developed method has been used to analyse serum samples from 18 prediabetic (PreDiab group) and 24 T2DM patients (T2DM group) from 1000PLUS cohort.

In the study performed by Jensen-Kroll et al., the effects of enriched galactooligosaccharides (GOS) and milk oligosaccharides (MOS) mixtures from caprine and bovine milk (consisting of mainly 6′-galactosyllactose, 3′-sialyllactose, and 6′-sialyllactose) on Caco-2 cells were investigated, and the treatment-specific metabolomes were described. The metabolomics workflow with pathway enrichment was established, which specifically addresses DI-FT-ICR-MS analyses and includes adaptations in terms of measurement technology and sample matrices. As a result, it was shown that MOS and GOS containing treatments can exert microbiome-independent effects on the metabolome of Caco-2 cells.

Shaver et al. have used the model system *C. elegans* to demonstrate that an augmented design combined with experimental blocks and other metabolomic QC approaches can be used to anchor studies and enable comparisons of stable spectral features across time without the need for compound identification.

Barrero-Rodríguez have presented TurboPutative (https://proteomics.cnic.es/TurboPutative/), a flexible and user-friendly web-based platform composed of four modules (Tagger, REname, RowMerger, and TPMetrics) that streamlines data handling, classification, and interpretability of untargeted LC-MS-based metabolomics data. The platform constitutes a promising and useful tool for the metabolomics community to speed up the arduous task of manual data curation that is required in the first steps of metabolite identification, improving the generation of biological knowledge.

Traquete et al. have proposed a novel role for mass-difference networks in untargeted metabolomics data analysis. The use of these networks as graphs for metabolic signatures and class discrimination have been demonstrated. Furthermore, two new metrics which illustrate how the graph properties of mass-difference networks can highlight the aspects of the information contained in data that are complementary to the information extracted from intensity-based data analysis have been proposed.

Małachowska et al. have reported the results of the study aimed to identify serum metabolic changes caused by an episode of diabetes ketoacidosis (DKA) and hypoglycemia (HG) that may indicate the mechanisms contributing to long-term consequences caused by these complications of diabetes. Eight metabolites whose levels may be traced in the serum, indicating the DKA or HG episode for up to 72 h and 48 h, have been found. The obtained results suggest that acute complications of diabetes may cause persistent metabolic disturbances long after pH and glucose level normalization.

Lackner et al. have established the workflow combining targeted isotopologue feature extraction with the non-targeted X^13^CMS routine. Metabolites, detected by X^13^CMS as differentially labeled between two biological conditions are subsequently integrated into the original targeted library. This workflow has been demonstrated in a PTEN (phosphatase and tensin homolog) null breast cancer cell line (MDA-MB-468) exploring metabolic pathway activities in the absence and presence of the selective PI3Kβ inhibitor AZD8186.

We would like to thank all the authors for their efforts and dedication in contributing their results to this special issue. They have all contributed to the advancement of the field of non-targeted metabolomics. We would also like to thank all the reviewers who have selflessly given their time and effort to produce a collection of articles of great interest and quality.

We are delighted to offer this collection of innovative articles, which provide a broad and in-depth perspective on the state of the art in the different stages of the non-targeted metabolomics workflow. In this exciting field not everything has been written yet, and we hope in the future to see more and better advances in methodology, which will allow us to standardise the process of obtaining more and better metabolomic information, with more throughput and productivity.
